# Photon-counting detector CT-based virtual monoenergetic reconstructions: repeatability and reproducibility of radiomics features of an organic phantom and human myocardium

**DOI:** 10.1186/s41747-023-00371-8

**Published:** 2023-10-25

**Authors:** Elias V. Wolf, Lukas Müller, U. Joseph Schoepf, Nicola Fink, Joseph P. Griffith, Emese Zsarnoczay, Dhiraj Baruah, Pal Suranyi, Ismael M. Kabakus, Moritz C. Halfmann, Tilman Emrich, Akos Varga-Szemes, Jim O‘Doherty

**Affiliations:** 1 Department of Diagnostic and Interventional Radiology, University Medical Center of the Johannes Gutenberg-University, Mainz, Germany; 2 Division of Cardiovascular Imaging, Department of Radiology and Radiological Science, Medical University of South Carolina, Charleston, SC USA; 3 Department of Radiology, University Hospital, LMU Munich, Munich, Germany; 4 Medical Imaging Centre, Semmelweis University, Budapest, Hungary; 5 German Centre for Cardiovascular Research, Partner site Rhine-Main, Mainz, Germany; 6 Siemens Medical Solutions USA Inc, Malvern, PA USA

**Keywords:** Myocardium, Phantoms (imaging), Radiomics, Reproducibility of results, Tomography (x-ray, computed)

## Abstract

**Background:**

Photon-counting detector computed tomography (PCD-CT) may influence imaging characteristics for various clinical conditions due to higher signal and contrast-to-noise ratio in virtual monoenergetic images (VMI). Radiomics analysis relies on quantification of image characteristics. We evaluated the impact of different VMI reconstructions on radiomic features in *in vitro* and *in vivo* PCD-CT datasets.

**Methods:**

An organic phantom consisting of twelve samples (four oranges, four onions, and four apples) was scanned five times. Twenty-three patients who had undergone coronary computed tomography angiography on a first generation PCD-CT system with the same image acquisitions were analyzed. VMIs were reconstructed at 6 keV levels (40, 55, 70, 90, 120, and 190 keV). The phantoms and the patients’ left ventricular myocardium (LVM) were segmented for all reconstructions. Ninety-three original radiomic features were extracted. Repeatability and reproducibility were evaluated through intraclass correlations coefficient (ICC) and *post hoc* paired samples ANOVA *t* test.

**Results:**

There was excellent repeatability for radiomic features in phantom scans (all ICC = 1.00). Among all VMIs, 36/93 radiomic features (38.7%) in apples, 28/93 (30.1%) in oranges, and 33/93 (35.5%) in onions were not significantly different. For LVM, the percentage of stable features was high between VMIs ≥ 90 keV (90 *versus* 120 keV, 77.4%; 90 *versus* 190 keV, 83.9%; 120 *versus* 190 keV, 89.3%), while comparison to lower VMI levels led to fewer reproducible features (40 *versus* 55 keV, 8.6%).

**Conclusions:**

VMI levels influence the stability of radiomic features in an organic phantom and patients’ LVM; stability decreases considerably below 90 keV.

**Relevance statement:**

Spectral reconstructions significantly influence radiomic features *in vitro* and *in vivo*, necessitating standardization and careful attention to these reconstruction parameters before clinical implementation.

**Key points:**

• Radiomic features have an excellent repeatability within the same PCD-CT acquisition and reconstruction.

• Differences in VMI lead to decreased reproducibility for radiomic features.

• VMI ≥ 90 keV increased the reproducibility of the radiomic features.

**Graphical Abstract:**

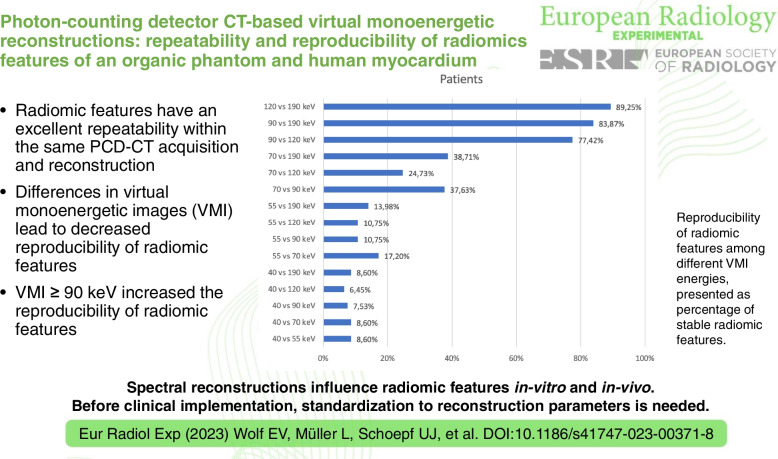

**Supplementary Information:**

The online version contains supplementary material available at 10.1186/s41747-023-00371-8.

## Background

Radiomics is a field of medical imaging that involves the extraction and analysis of quantitative features from medical images, such as computed tomography (CT), magnetic resonance imaging, or positron emission tomography [1, 2]. In combination with machine learning algorithms, radiomics data can be used to provide inferences about tumor characteristics and correlate them with tumor histopathology [3, 4]. Furthermore, radiomic features can be used as biomarkers in oncologic treatment for improved accuracy of prognosis [5–7] and better outcome prediction [8–10], based on machine learning classification models.

In conventional CT imaging, only one image is traditionally formed, which is generated as the average energy of the x-ray spectrum. Dual energy CT (DECT), however, utilizes data acquired at two distinct energy levels to create images that highlight different tissue attenuation properties. The generation of other DECT specific image datasets such as virtual monoenergetic images (VMI), generated at specific energies in the x-ray spectrum) enable the reduction of beam-hardening artifacts and the improvement of the contrast-to-noise ratio [11]. However, such advanced reconstruction was rarely used for cardiac applications due to the limited temporal resolution of the DECT systems. With the recent introduction of a first-generation clinical photon-counting detector CT (PCD-CT) system, spectral imaging based VMI datasets are available by default, without the temporal resolution penalty.

The influence of VMI on the repeatability and reproducibility of radiomic features on energy-integrating detector (EID)-based DECT has previously been investigated [12]. Euler et al. [12] demonstrated in organic phantoms a high repeatability when CT scan settings and reconstructions were kept constant. However, the reproducibility decreased with a larger difference between the two VMI reconstructions being compared. The influence of different VMI has also previously been explored in oncology, including for cervical lymphadenopathy [13, 14], parotid tumors [15], pancreatic tumors [16], and liver lesions [12]. Accordingly, the refinement of VMI could improve the detection and evaluation of several malignant lesions.

Therefore, the aim of this study was to evaluate the impact of different VMI reconstructions on radiomic features in *in vitro* and *in vivo* PCD-CT datasets.

## Methods

### Phantom study

**Fig. 1:**
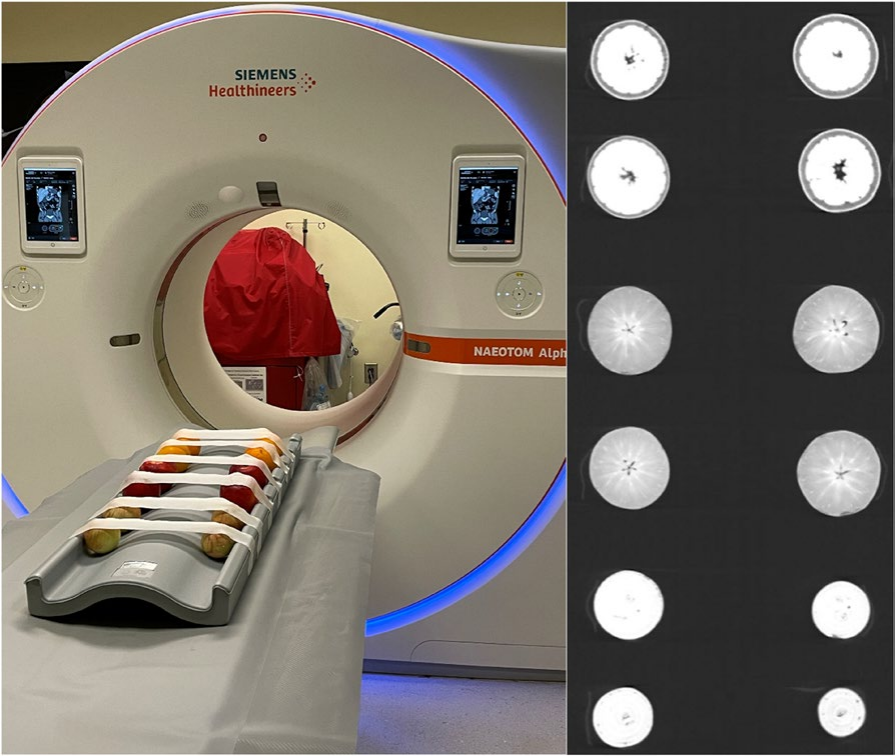
Phantom setup (left) and a cross-sectional PCD-CT image (right) of the fruit samples reconstructed at 70 keV (window level W1200/C-600)

In order to verify consistency and stability in our radiomic analysis pipeline, phantom experiments were conducted prior to clinical studies. The phantom consisted of twelve organic phantoms (four oranges, four onions, and four apples). Four organic samples of the same item were used to verify radiomic feature extraction repeatability and to identify errors that may be associated with analysis of a specific feature of interest. All objects were placed and fixed on a radiolucent stretcher prior to scanning (Fig. 1).

### Phantom data acquisition and reconstruction parameters

Phantom images were acquired on a PCD-CT system (NAEOTOM Alpha, Siemens Healthineers, Forchheim, Germany). The two photon-counting cadmium telluride, CdTe, detectors in the PCD-CT system, each with a nominal 144 × 0.4 mm collimation, enable the acquisition of spectral CT data with a maximum temporal resolution of 66 ms. A standard coronary CT angiography (CCTA) protocol was used in sequential mode with a slice thickness of 0.6 mm, increment of 0.6 mm, and a manually set tube voltage of 120 kVp. Tube current was set to 120 mAs with a resulting volume CT dose index (CTDI_vol_) of 9.45 mGy. A signal mimicking an electrocardiogram was generated to reflect a heart rate of 60 beats per minute, and the examination was initiated during the diastolic phase (75% of the cardiac cycle). All twelve organic phantoms were measured five times to account for the scanner’s variability, each time shifting by approximately 2 mm and rotating by approximately 2° [17].

Images were reconstructed using a quantitative reconstruction kernel (Qr36), quantum iterative reconstruction (QIR) level of 3, a matrix size of 512 with a reconstructed field of view of 750 mm × 200 mm (so that all samples were in the same field of view), and a slice thickness of 0.6 mm. VMIs were reconstructed at six different energies (40, 55, 70, 90, 120, and 190 keV). Detailed acquisition and reconstruction parameters are shown in Table 1.

**Table 1:** PCD-CT acquisition and reconstruction parameters

	Phantoms	Patients
Tube potential (kVp)	120	120
Quantum Iterative Reconstruction level	3	3
Reconstruction kernel	Qr36	Qr36
Slice thickness/increment (mm)	0.6/0.6	0.6/0.6
Field of view (mm)	750 × 250	200 × 200
Matrix size	512 × 512	512 × 512
Monoenergetic levels (keV)	40, 55, 70, 90, 120, and 190	40, 55, 70, 90, 120, and 190

### Patient study

Patient data used in this study was acquired as part of a larger prospective study evaluating PCD-CT for cardiovascular applications. All participants gave written informed consent, and the study protocol was approved by the local Institutional Review Board in accordance with the HIPAA guidelines. Patients were enrolled for this research study based on the following inclusion criteria: (1) individuals who underwent electrocardiographically gated cardiac imaging due to a clinical indication of chest pain; (2) age above 18 years old; (3) no allergy to iodine-based contrast media; (4) intact kidney function with a glomerular filtration rate higher than 45 mL/min/m^2^; (5) not currently pregnant or lactating; and (6) able to provide consent. Patients who did not meet all of the inclusion criteria or refused consent were excluded from the study.

### Patient data acquisition and image reconstruction

The phantom data acquisition parameters were matched to the acquired patient CCTA scans. A sequential cardiac protocol was used for all CCTA scans, which included ECG triggering and a triphasic contrast injection protocol. This involved injecting an initial bolus of nonionic iodinated contrast agent (50 mL, iopromide 350 mgI/ml; Ultravist, Bayer, Leverkusen, Germany), followed by a 50% mixture of contrast and saline (20 mL), and a saline chaser (25 mL). The injection rate was consistent for all three phases (4 mL/s). If it was not clinically contraindicated, patients were given 0.4 mg nitroglycerin approximately 5 min before the scan, and those with heart rates above 70 beats per minute received 5 mg metoprolol intravenously.

Reconstruction parameters were also identical to the phantom experiment, except for the reduced field of view (200 × 200 mm) limited to the heart.

### Image analysis

Digital Imaging and Communications in Medicine, DICOM, images from phantom and patient scans were analyzed using a single radiomic analysis pipeline. Both sets of data were reformatted with 5.0-mm slice thickness and 5.0-mm increment, as is common process in radiomic analysis [18, 19]. The phantom images were loaded into an open-source software (3D Slicer, Version 4.11).

The organic phantoms were semiautomatically segmented with a grow-from-seeds algorithm for each sample group at the 70 keV level. This algorithm was chosen for the phantom images to ensure an accurate and easy workflow. A three-dimensional volume of interest (VOI) of the organic phantoms was generated after applying the algorithm. Each sample was analyzed within their three groups (apples, oranges, and onions). All segmentations were performed on the 70 keV image series, and the VOIs were transferred to the remaining image series (40, 55, 90, 120, and 190 keV). The same procedure was performed for the other four repeated scans.

**Fig. 2:**
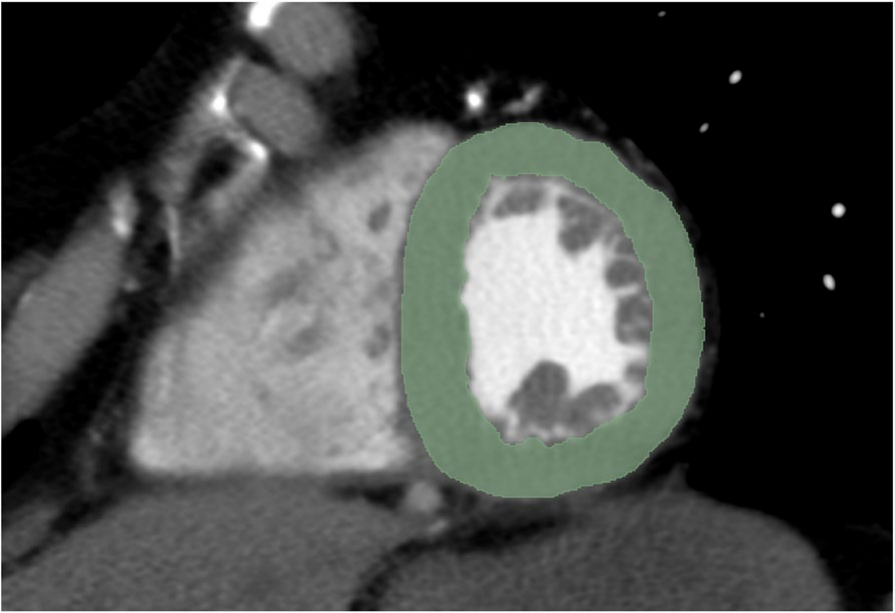
Representative example of myocardial segmentation in a patient PCD-CT scan at 70 keV

Additionally, the patient scans were re-orientated on a short-axis view of the left ventricle and simultaneously reformatted according to the phantom study. The six VMI datasets were imported into 3D Slicer. The left ventricular myocardium (LVM) was manually segmented in single slice at the middle of the short-axis stack. The segmented area excluded the trabeculations and papillary muscles (Fig. 2). Segmentation was performed by a reader with 2 years of experience in cardiovascular radiology under the supervision of a board-certified cardiovascular radiologist with 12 years of experience. As in the phantom data, segmentations were performed on the 70 keV image, and the resulting VOIs were transferred to the other five VMI datasets.

### Feature extraction

For extraction, a total of 93 radiomic features were generated in an open source-software (SlicerRadiomics, Pyradiomics, version 3.0.1) with the following feature classes specific to the analysis software: First Order (FO), Gray Level Co-occurrence Matrix (GLCM), Gray Level Dependence Matrix (GLDM), Gray Level Run Length Matrix (GLRLM), Gray Level Size Zone Matrix (GLSZM), and Neighboring Gray Tone Difference Matrix-Features (NGTDM). A standard output from Pyradiomics provides a total of 107 radiomic features. The feature class shape (14 features) was excluded in this analysis because all shape parameters were identical through the transfer from the 70 keV level segmentation to the remaining reconstructions. A full mathematical description of these feature families and representative features within them is available in the software documentation [20]. All 93 radiomic features were extracted for the six different VMIs. From all the segmentations in the phantoms and patients, we analyzed a total of 21,204 radiomic features. The normalized feature values between the range 0 and 1 were calculated by:

$${F}_{n}\left[i\right]= \frac{F\left[i\right]-{F}_{min}}{{F}_{\mathit{max}}- {F}_{min}}$$

where F[i], Fmin and Fmax represent measured, minimum and maximum value of radiomic feature respectively. The normalized radiomic features were analyzed in their sample groups for the five repeated scans.

### Data analysis of repeatability and reproducibility

Radiomic features were considered stable if *post hoc* tests from univariate analysis of variance did not reveal significant differences between VMIs. Repeatability was defined as feature stability between five repeated phantom scans with the same reconstruction settings. While reproducibility was the feature stability calculated between different VMIs in the same scan (phantom or patient, respectively). The percentage of stable radiomic features of all 93 tested features was used for reporting repeatability or reproducibility.

Furthermore, for reproducibility, the percentage deviation of the normalized values from different VMIs were compared to the normalized values from the lowest VMI (40 keV) in our study.

From previous literature, twelve radiomic features have demonstrated stability between VMIs ranging from 40 to 120 keV on PCD-CT scans between different organs (firstorder_Entropy, glcm_Differenceentropy, glcm_Jointentropy, gldm_DependenceEntropy, gldm_DependenceNonUniformity, gldm_DependenceNonUniformityNormalized, gldm_GrayLevelNonUniformity, glrlm_GrayLevelNonUniformity, glrlm_RunLengthNonUniformity, glrlm_RunLengthNonUniformityNormalized, glrlm_ShortRunEmphasis, ngtdm_Coarseness) [21]. We also specifically examined these preselected features according to our phantom study via intraclass correlation coefficients (ICC) calculations.

### Statistical analysis

Statistical analysis was performed with software packages (SPSS Statistics, version 21.0, IBM Corp Armonk, NY; R, version 4.2.2, The R Foundation for Statistical Computing, Vienna, Austria with RStudio, 2022.07.2, Boston, MA, USA with the packages tidyverse [22] and ggplot2 [23], and Excel, version 16.64, Microsoft Corporation, Redmond, WA). For normally distributed data, tested with the Kolmogorov-Smirnov test, mean ± standard deviation was utilized, while for non-normally distributed data, median with interquartile range was utilized. Variables classified as categorical were presented as frequencies and proportions. The difference between radiomic features were compared with a paired samples ANOVA *t*-test and the corresponding post hoc tests. A *p* value ≤ 0.05 was considered as statistically significant difference and therefore interpreted as unstable feature between different VMIs. Two-way mixed ICC was used to assess the degree of agreement between distinct VMIs, and the agreement was interpreted as follows: ≤ 0.39, poor agreement; 0.4 to 0.59, moderate agreement; 0.6 to 0.74, good agreement; and ≥ 0.75 excellent agreement [24, 25].

## Results

### Phantom repeatability analysis

**Fig. 3:**
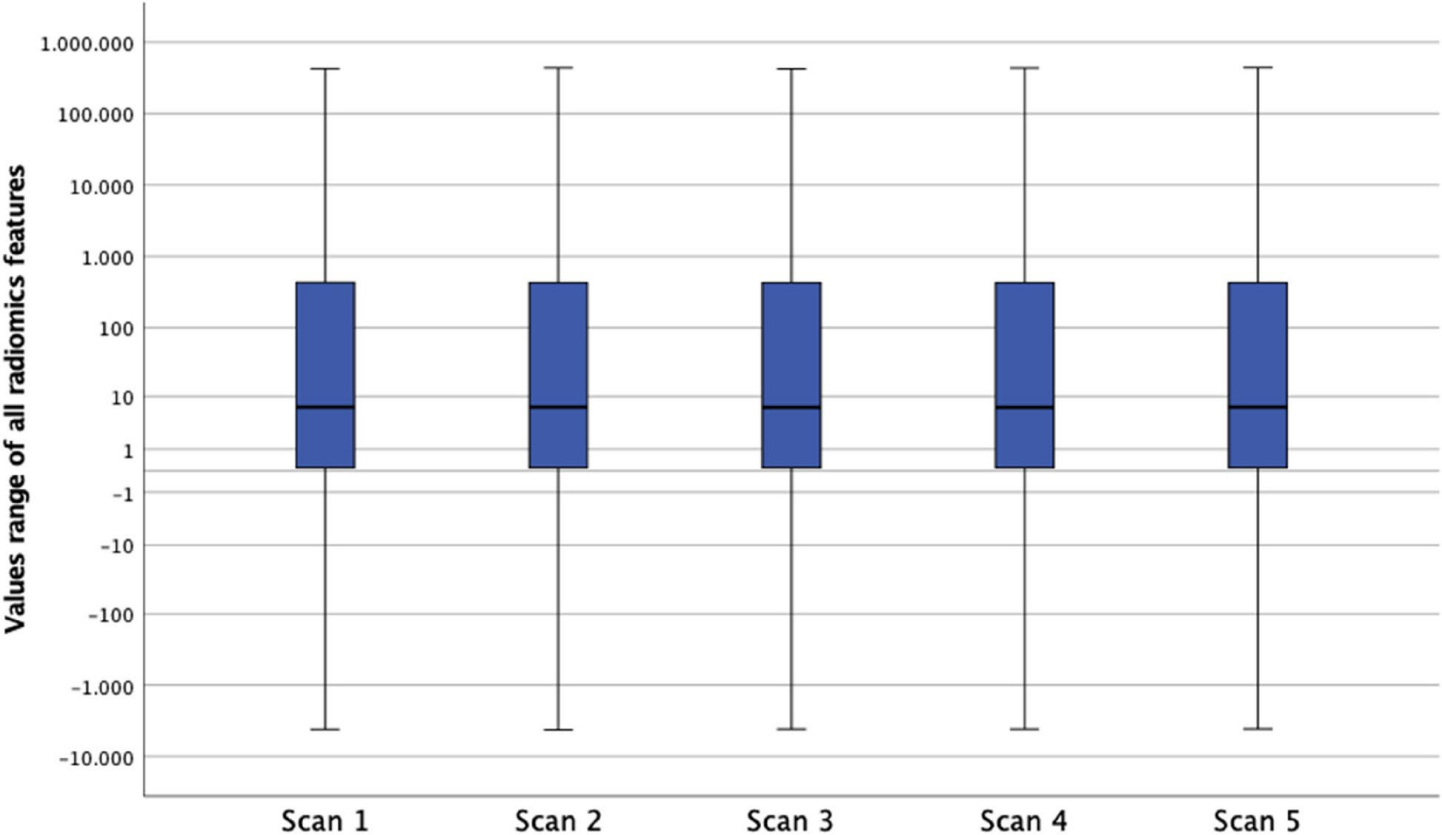
Logarithmically scaled box plots showing repeatability of radiomic features between the five phantom scans

In a logarithmic boxplot, all 93 radiomic features from the three different organic phantoms and five repeated scans showed no significant differences to each other (Fig. 3). Respective ICCs across all VMIs yielded excellent values in repeatability between the different scans (apples ICC = 1.00, oranges ICC = 1.00, and onions ICC = 1.00). Additionally, the repeatability for the 70 keV VMI for all three samples over the five repeated scans is shown through scatter plots (Supplemental Figure S1).

### Phantom reproducibility analysis

**Fig. 4:**
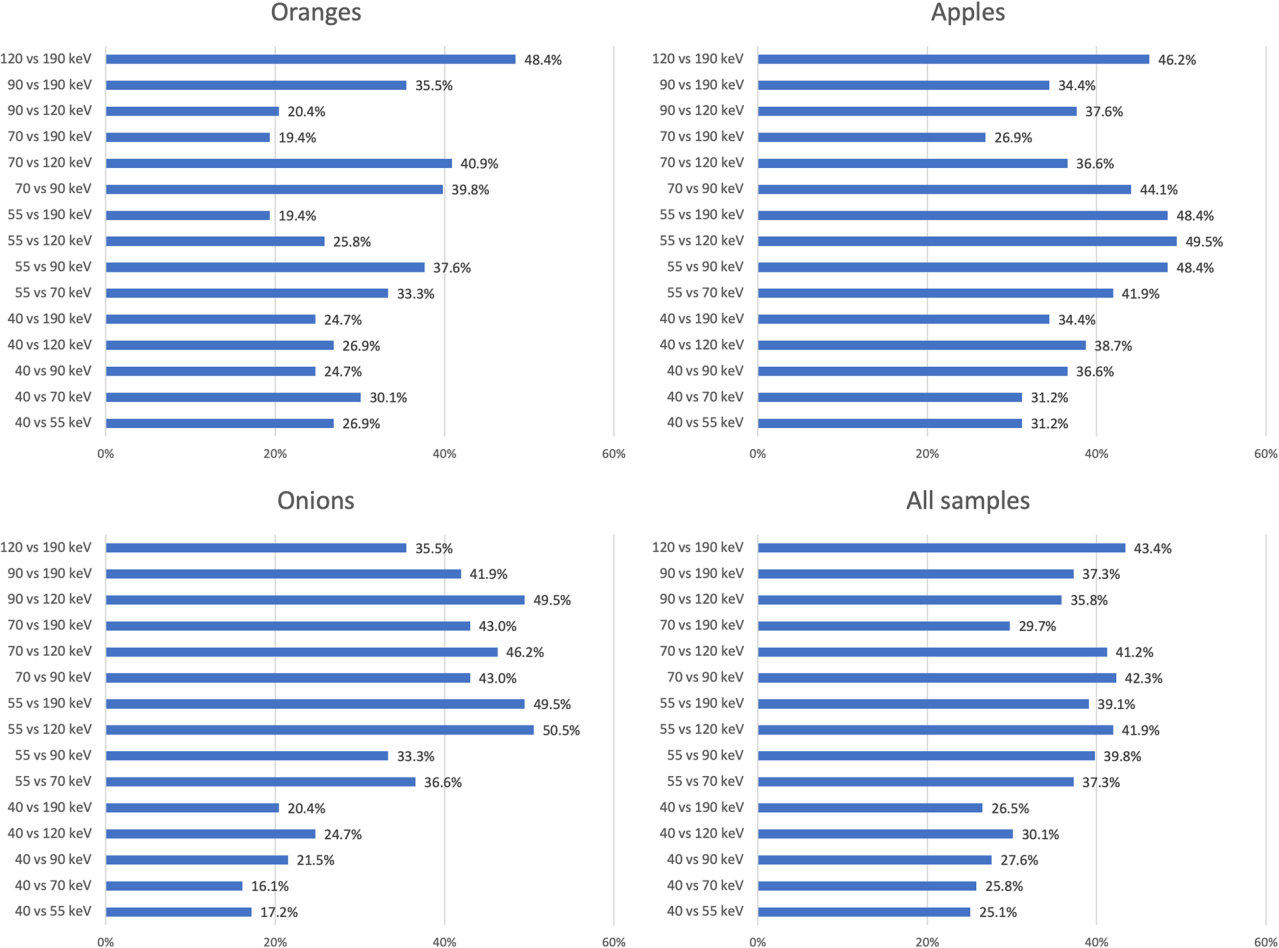
Reproducibility of radiomic features between different VMI energies (40, 55, 70, 90, 120, and 190 keV) in oranges, onions, apples, and all samples. The values are shown as percentage of stable radiomic features out of all 93 features

The different samples did not show a substantial reproducibility among the different VMIs (mean reproducibility of all samples: 34.9 %, Fig. 4). The highest values found with 49.5% (apples, 55 *versus* 120 keV), 48.4% (oranges, 120 *versus* 190 keV), and 50.5% (onions, 55 *versus* 120 keV). Additionally, the reproducibility compared to the 40 keV VMI for all three samples is shown in scatter plots (Supplemental Figure S2).

### Preselected features reproducibility analysis

The previous described twelve stable radiomic features were tested for reproducibility between six different VMIs on the organic phantom. Excellent agreements with ICC values above 0.75 were seen in comparisons with both VMI ≥ 90 keV. Comparisons of 90 *versus* 120 keV (apples = 11 (91.7%), oranges = 10 (83.3%), onions = 11 (91.7%)), 90 *versus* 190 keV (apples = 11 (91.7%), oranges = 10 (83.3%), onions = 6 (50.0%)), and 120 *versus* 190 keV (apples = 11 (91.7%), oranges = 11 (91.7%), onions = 9 (75.0%)) showed excellent (≥ 0.75) ICC values of the twelve preselected features.

At VMIs ≥ 90 keV (90 *versus* 120 keV, 90 *versus* 190 keV, and 120 *versus* 190 keV), 91.7% of radiomic features were stable, whereas below 90 keV (40 *versus* 70 keV, 40 *versus* 90 keV, 40 *versus* 120 keV, and 40 *versus* 190 keV), only 8.3% were stable. This illustrates a trend that a higher percentage of radiomic features was stable at higher VMIs (≥ 90 keV) and VMIs in close proximity of each other (*i.e.*, 90 *versus* 120 keV: 32/36, 88.9%) than at VMIs with a greater keV difference (*i.e.*, 40 *versus* 120 keV: 4/36, 11.1%). The summation/percentage values (Table 2) and the individual values (Supplemental Table S1) are shown in the corresponding tables.

**Table 2:** ICC > 0.75 summation from 12 previously described radiomics features

	40 vs 55	40 vs 70	40 vs 90	40 vs 120	40 vs 190	55 vs 70	55 vs 90	55 vs 120	55 vs 190	70 vs 90	70 vs 120	70 vs 190	90 vs 120	90 vs 190	120 vs 190
Apples	8 (66.7%)	1 (8.3%)	1 (8.3%)	1 (8.3%)	1 (8.3%)	4 (33.3%)	4 (33.3%)	3 (25.0%)	4 (33.3%)	9 (75.0%)	9 (75.0%)	9 (75.0%)	11 (91.7%)	11 (91.7%)	11 (91.7%)
Oranges	6 (50.0%)	4 (33.3%)	2 (16.7%)	2 (16.7%)	3 (25.0%)	4 (33.3%)	3 (25.0%)	2 (16.7%)	4 (33.3%)	7 (58.3%)	7 (58.3%)	9 (75.0%)	10 (83.3%)	10 (83.3%)	11 (91.7%)
Onions	7 (58.3%)	7 (58.3%)	7 (58.3%)	1 (8.3%)	2 (16.7%)	7 (58.3%)	8 (66.7%)	4 (33.3%)	4 (33.3%)	8 (66.7%)	2 (16.7%)	3 (25.0%)	11 (91.7%)	6 (50.0%)	9 (75.0%)
Total	21 (58.3%)	12 (33.3%)	10 (27.8%)	4 (11.1%)	6 (16.7%)	15 (41.7%)	15 (41.7%)	9 (25.0%)	12 (33.3%)	24 (66.7%)	18 (50.0%)	21 (58.3%)	32 (88.9%)	27 (75.0%)	31 (86.1%)

### Myocardium reproducibility analysis

The study population included a total of 23 patients (16 men, 69.6 %) with an average age of 65 ± 12 years (mean ± standard deviation). The radiation dose of the CCTA scan had a median CTDI_vol_ of 24.0 (interquartile range 17.7−50.4) mGy. Details of the study population are given in Table 3.

**Table 3:** Patient characteristics

*N*	23
Female (%)	7 (30.4)
Age (years)	65 ± 12.1
BMI (kg/m^2^)	30.1 ± 7.1
Heart rate (bpm)	65.3 ± 10.8
CTDI_vol_ (mGy)	24.0 (17.7–50.4)
DLP (mGy*cm)	557.5 ± 384.7

*BMI* Body mass index, *bpm* Beats per minute, *CTDI* Computer tomography dose index, *DLP* Dose length product, *PCD-CT* Photon-counting detector CT

Values are mean ± standard deviation, median (interquartile range), *n* (frequencies)

**Fig. 5:**
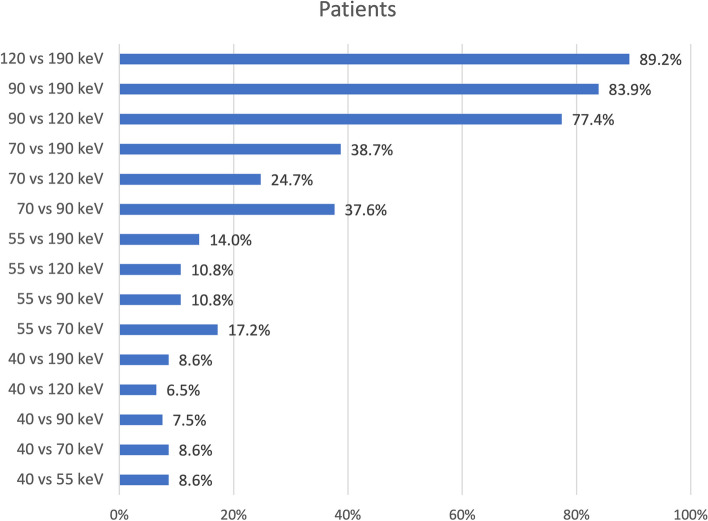
Reproducibility of radiomic features among different VMI energies (40, 55, 70, 90, 120, and 190 keV) in a single myocardial slice in patients. The values are shown as percentage of stable radiomic features out of all 93 features

The patient cohort also showed a higher percentage of stable radiomic features between VMI energies comparisons ≥ 90 keV (90 *versus* 120 keV, 77.4%¸ 90 *versus* 190 keV, 83.9%; and 120 *versus* 190 keV, 89.3%). This trend decreased with lower VMIs down to 6.5% (40 *versus* 120 keV, Fig. 5). Close proximity of VMIs did not always lead to a higher ICC result (*e.g.*, 40 *versus* 55 keV, 8.6%). Interestingly, the lowest VMI at 40 keV resulted in the lowest correlation values, all under 10% (average 8.0%).

## Discussion

This study investigated the difference in radiomic features derived from various VMI reconstructions in an organic phantom and a patient cohort scanned on a PCD-CT system. The major findings were the following. First, radiomic features had an excellent repeatability (ICC = 1.00) in organic phantoms when using the same acquisition and reconstruction parameters. Second, while the comparison of different VMIs showed inconsistent reproducibility of radiomic features in the organic phantom samples, there was a higher reproducibility between higher VMIs (90, 120, and 190 keV) in twelve preselected radiomic features, as well as in the patient cohort. Third, VMIs < 90 keV result in decreased ICC values, which should be considered while using radiomic features. The knowledge gained from these findings could guide VMI choice in further clinical studies of radiomic features.

By demonstrating almost perfect repeatability of radiomic features within the same VMI, our phantom study indicates that radiomic features are reliable when using the same acquisition and reconstruction parameters. Conversely, reproducibility was low between different VMIs in both the phantom and patient measurements. This finding implies that slight modification of the VMI impacts the characteristics of radiomic features. Furthermore, there was no transferability of the same trend from the phantom to patient’s results. A possible reason could be the difference in textures between the organic phantom and the myocardium, especially in the presence of contrast agent. Generally, the agreement between phantom and patient experiments is limited in the context of radiomic features and needs to be explored further. Interestingly, we obtained a stable plateau phase in our patient cohort between VMIs of 90 and 190 keV. A possible explanation would be that iodine, as a contrast agent, is an important factor that influences the radiomic features analysis. At VMIs ≥ 90 keV, there is minimal change in the attenuation coefficient of iodine [26], therefore improved reproducibility of the radiomic features may be observed. On the other hand, this study observed the instability of radiomic features at lower VMIs. At lower VMIs the gap to the attenuation coefficient of iodine is closer and differences in VMIs may have a greater impact on the stability of radiomic features.

Euler et al. [12] investigated the impact of different VMIs on the repeatability and reproducibility of radiomic features in a phantom on a DECT system. According to their results, there was a high repeatability of radiomic features within the same VMI. Repeatability was roughly 80% when acquisition and reconstruction parameters were kept constant. Similar to our study, the reproducibility of radiomic features differed between various VMIs. Increased reproducibility was found in reconstructions with lower keV differences. This trend was not evaluated in our phantom or patient study and could be attributed to several different factors, such as different statistical methods (concordance–correlation–coefficient *versus* ANOVA t-test), different selection of radiomic features (1218 *versus* 93) or different CT scanners (EID-CT *versus* PCD-CT). However, a common conclusion is that the use of different VMIs does not reproduce similar radiomic feature values on EID-CT or PCD-CT.

Mackin et al. [27] investigated inter-scanner variability of radiomic features generated by four different CT vendors. They conducted a phantom study consisting of 10 different materials imaged in 17 different scan modes. The results showed that the variability of radiomic features depended not only on the scanner but also on the acquisition and reconstruction parameters. This study highlights the importance of consistent image settings for the reproducibility of radiomic features on EID-CT, which is supported by other similar studies in the literature [28–33].

While most image settings need to remain constant to achieve high radiomic feature reproducibility, other settings such as tube current can be changed without substantial effect of radiomic feature values. An organic phantom study by Hertel et al. [34] demonstrated a test-retest stability of 75% of the radiomic features between different radiation dose values (10 mAs, 50 mAs, and 100 mAs). Their study demonstrated that reproducibility of radiomic features was not substantially affected by changes in radiation dose.

Conversely, when image settings such as kernel or spatial resolution change, there is a fundamental decrease in radiomic feature reproducibility. For example, Dunning et al. [35] demonstrated that 13 of 14 relevant radiomic features differed by more than 50% between PCD- and EID-CT. Further, Tharmaseelan et al. [21] demonstrated that VMI (40–120 keV in 10 steps) has an important impact on radiomic feature stability in various abdominal tissue (liver, lung, spleen, psoas muscle and subcutaneous fat). Of the 93 features analyzed in their study, only 12 had ICC values ≥ 0.75 in three or more organs. Additionally, different tissues had different features with the best reproducibility. Interestingly, the twelve radiomic features with the highest agreement found in the study by Tharmaseelan et al. [21] did not have high reproducibility in our study. Perhaps this difference was due to different acquisition settings or content of the phantom/tissue being imaged. Overall, understanding the effect of each imaging settings on the reproducibility of radiomic features is critical to gathering information that is ultimately useful in patient management.

Clinical application and clinical decision models may potentially involve radiomic feature assessment in the future. Before the implementation of such models, it is crucial to have a better understanding of the stability of radiomic features and their influence by disruptive factors. Our investigation contributes to this knowledge by exploring the influence of VMIs on the stability of radiomic features in a phantom and in human LVM.

Several limitations should be taken into account when evaluating this investigation. First, a larger patient group would be beneficial in future studies, as our patient cohort was limited to 23 individuals. However, large cohort studies in radiomics are generally rare. Second, patient PCD-CT scans were acquired with a higher radiation dose levels than the phantom PCD-CT scans. Radiation dose was lower for organic phantom scans as we utilized a clinical protocol with automated dose modulation, which resulted in a lower value of mAs. Nevertheless, several aforementioned studies have shown that different radiation dose levels do not change radiomic feature values substantially [12, 35]. Third, the phantom texture materials differ from the myocardial texture with contrast enhanced scans, and therefore the results may not be transferable. Further investigations with native CT scans have to be investigated, which were not available in this study. An improved heart phantom model would be preferable. Fourth, possible variations in heart rate and other acquisition parameters, such as breath-hold and positioning, may have an impact on the patient measurements, but these potential effects were not explored in the study. Fifth, this study did not investigate the relationship between radiomic features and clinical outcomes, which should be considered in larger clinical studies. This investigation concentrates on the influence of VMIs on the stability of radiomic features. Sixth, intra- and inter-rater reproducibility was not included in the scope of this study and should be highlighted in further clinical investigations. Finally, we considered only examinations from PCD-CT, and these results may not be transferable to other CT scanners. Further studies are needed to evaluate the influence of different scanner types on radiomic features.

In conclusion, VMI influences *in vitro* and *in vivo* characteristics of radiomic features on PCD-CT, resulting in a different rate of reproducibility among different VMI. There seems to be a better correlation of reproducibility between higher VMI in myocardial texture. Further studies are necessary to determine imaging protocols for optimizing radiomic feature stability on PCD-CT.

### Supplementary Information

**Additional file 1: Supplementary Figure S1.** Scatter plot of all 93 normalized radiomics features for the five repeated scans at 70 keV.**Supplementary Figure 2.** Scatter plot of all 93 normalized radiomics features deviation compared to the related normalized radiomics features at 40 keV between -100 and 100%.** Supplementary Table S1.** ICC of 12 previous described stable features between the different VMIs (40, 55, 70, 90, 120, and 190 keV).

## Data Availability

The datasets used and/or analyzed during the current study are available from the corresponding author on reasonable request.

## Abbreviations

CCTA: Coronary CT angiography
CT: Computed tomography
CTDI_vol_: Volume CT dose index
DECT: Dual energy CT
EID: Energy-integrating detector
PCD-CT: Photon-counting detector CT
VMI: Virtual monoenergetic image
VOI: Volume of interest
