# Supplementary material for: Photon-counting detector CT-based virtual monoenergetic reconstructions: repeatability and reproducibility of radiomics features of an organic phantom and human myocardium
Source: Eur Radiol Exp. 2023 Oct 25;7:59. doi: 10.1186/s41747-023-00371-8 (PMC10597903; doi:10.1186/s41747-023-00371-8)
Supplement: Supplementary file 1 — Additional file 1: Supplementary Figure S1. Scatter plot of all 93 normalized radiomics features for the five repeated scans at 70 keV.Supplementary Figure 2. Scatter plot of all 93 normalized radiomics features deviation compared to the related normalized radiomics features at 40 keV between -100 and 100%. Supplementary Table S1. ICC of 12 previous described stable features between the different VMIs (40, 55, 70, 90, 120, and 190 keV). [file 41747_2023_371_MOESM1_ESM.pdf]

Photon-counting detector CT-based virtual monoenergetic reconstructions: repeatability and reproducibility of radiomics features of an organic phantom and human myocardium

ELECTRONIC SUPPLEMENTARY MATERIAL

Suppl. Figure 1: Scatter plot of all 93 normalized radiomics features for the five repeated scans at 70 keV.

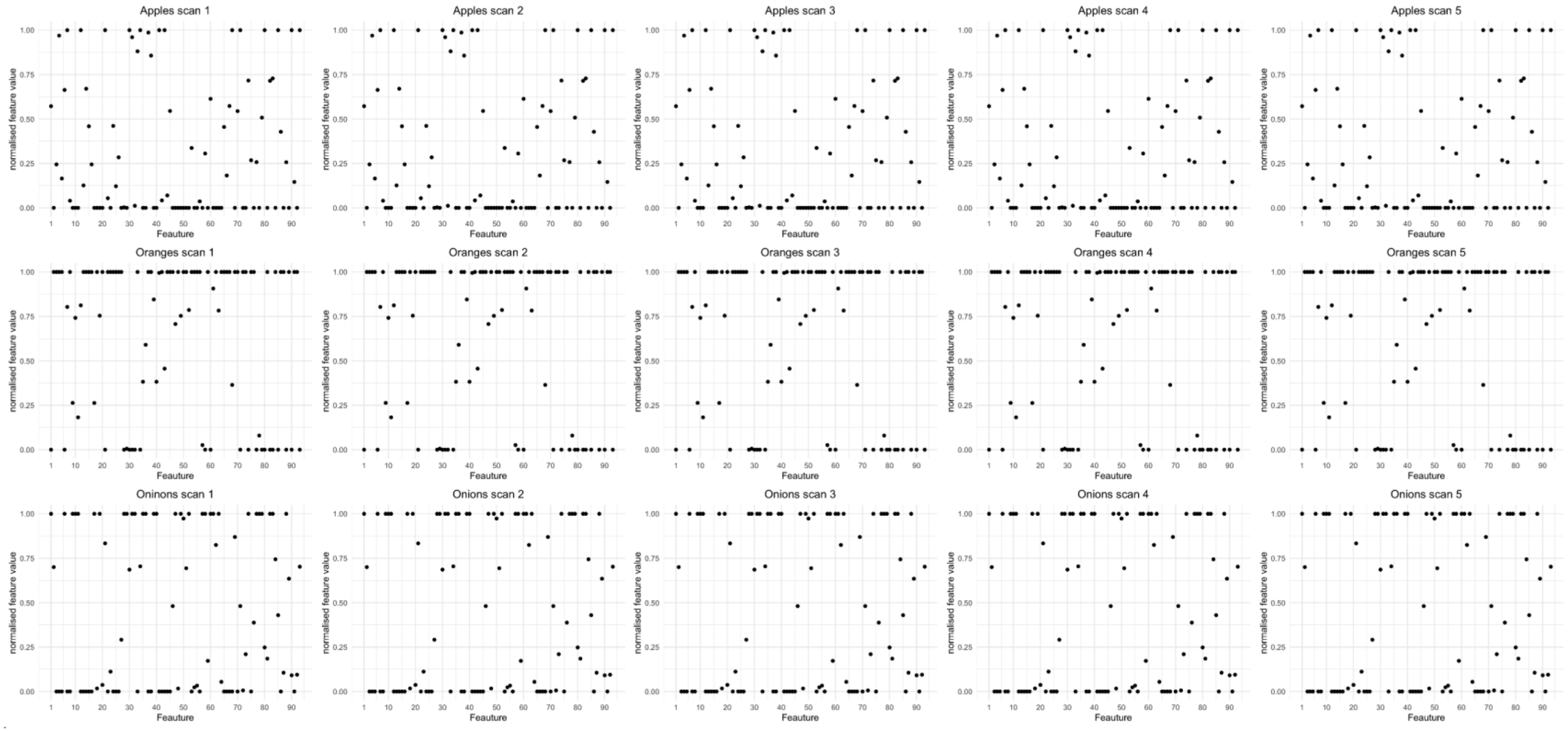

Suppl. Figure 2: Scatter plot of all 93 normalized radiomics features deviation compared to the related normalized radiomics features at 40 keV between -100 and 100 %.

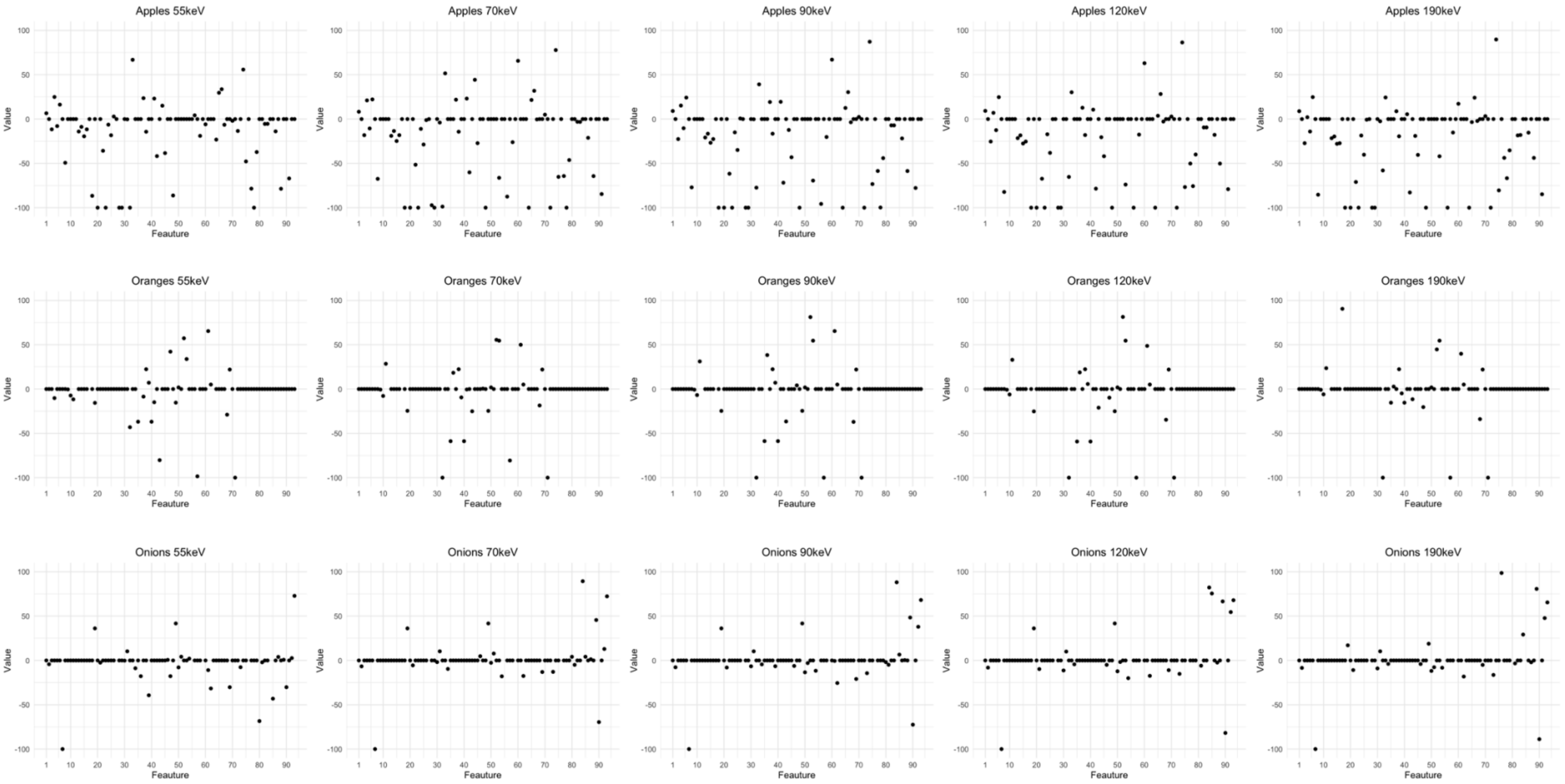

Suppl. Table 1: ICC of 12 previous described stable features between the different VMIs (40, 55, 70, 90, 120 and 190 keV)

Apples															
	40 vs 55	40 vs 70	40 vs 90	40 vs 120	40 vs 190	55 vs 70	55 vs 90	55 vs 120	55 vs 190	70 vs 90	70 vs 120	70 vs 190	90 vs 120	90 vs 190	120 vs 190
firstorder_Entropy	**0.778**	0.555	0.383	0.368	0.328	**0.944**	**0.866**	**0.835**	**0.810**	**0.983**	**0.967**	**0.954**	**0.997**	**0.992**	**0.999**
glcm_Differenceentropy	**0.989**	**0.979**	**0.968**	**0.967**	**0.961**	**0.996**	**0.989**	**0.984**	**0.977**	**0.997**	**0.994**	**0.990**	**0.999**	**0.996**	**0.999**
glcm_Jointentropy	**0.781**	0.610	0.506	0.495	0.456	**0.948**	**0.877**	**0.841**	**0.806**	**0.982**	**0.962**	**0.942**	**0.996**	**0.987**	**0.998**
gldm_DependenceEntropy	**0.795**	0.290	/	/	/	0.653	0.133	/	0.191	**0.930**	**0.890**	**0.923**	**0.986**	**0.985**	**0.992**
gldm_DependenceNonUniformity	**0.926**	0.467	0.235	/	/	0.484	0.115	/	/	0.399	0.084	0.164	**0.927**	**0.972**	**0.986**
gldm_DependenceNonUniformityNormalized	**0.862**	/	/	/	/	/	/	/	/	/	/	/	**0.915**	**0.965**	**0.984**
gldm_GrayLevelNonUniformity	0.740	0.225	/	/	/	0.838	0.581	0.485	0.406	**0.949**	**0.911**	**0.878**	**0.993**	**0.983**	**0.997**
glrlm_GrayLevelNonUniformity	0.733	0.223	/	/	/	0.737	0.393	0.279	0.264	**0.937**	**0.888**	**0.870**	**0.988**	**0.978**	**0.998**

glrlm_RunLengthNonUniformity	**0.918**	0.695	0.500	0.210	0.437	**0.919**	**0.768**	0.607	**0.751**	**0.907**	**0.835**	**0.907**	**0.986**	**0.993**	**0.981**
glrlm_RunLengthNonUniformityNormalized	**0.782**	0.321	/	/	/	0.653	0.179	/	0.065	**0.879**	**0.756**	**0.850**	**0.977**	**0.990**	**0.977**
glrlm_ShortRunEmphasis	0.701	0.288	/	/	/	0.515	/	/	/	**0.899**	**0.788**	**0.896**	**0.979**	**0.988**	**0.970**
ngtdm_Coarseness	/	/	/	/	/	/	/	/	/	/	/	/	/	/	/
Total	*0.750*	*0.423*	*0.432*	*0.408*	*0.436*	*0.699*	*0.490*	*0.576*	*0.474*	*0.806*	*0.734*	*0.761*	*0.895*	*0.902*	*0.907*
Oranges															
	40 vs 55	40 vs 70	40 vs 90	40 vs 120	40 vs 190	55 vs 70	55 vs 90	55 vs 120	55 vs 190	70 vs 90	70 vs 120	70 vs 190	90 vs 120	90 vs 190	120 vs 190
firstorder_Entropy	0.685	**0.800**	0.468	**0.769**	**0.777**	**0.828**	0.745	0.723	**0.763**	**0.900**	**0.948**	**0.959**	**0.946**	**0.943**	**0.996**
glcm_Differenceentropy	**0.965**	**0.989**	**0.812**	0.745	**0.830**	**0.965**	**0.902**	**0.853**	**0.911**	**0.869**	**0.809**	**0.884**	**0.994**	**0.988**	**0.975**
glcm_Jointentropy	**0.802**	**0.837**	0.361	0.650	0.709	**0.807**	0.669	0.672	**0.786**	0.617	**0.855**	**0.939**	**0.924**	**0.877**	**0.963**
gldm_DependenceEntropy	0.712	0.347	/	/	/	0.131	0.553	0.523	0.617	0.357	0.511	0.623	**0.982**	**0.956**	**0.990**
gldm_DependenceNonUniformity	**0.773**	0.556	0.488	0.496	0.536	0.420	0.623	0.611	0.653	**0.810**	**0.785**	**0.823**	**0.998**	**0.997**	**0.997**

gldm_DependenceNonUniformityNormalized	0.658	0.315	0.316	0.328	0.370	0.125	0.498	0.479	0.529	**0.759**	0.725	**0.772**	**0.997**	**0.997**	**0.996**
gldm_GrayLevelNonUniformity	/	0.076	/	0.416	0.688	0.056	**0.769**	0.163	/	/	/	0.399	0.335	/	**0.901**
glrlm_GrayLevelNonUniformity	0.221	/	0.322	0.303	0.310	/	0.136	0.040	0.059	0.740	0.723	**0.835**	**0.996**	**0.988**	**0.983**
glrlm_RunLengthNonUniformity	**0.925**	**0.886**	**0.896**	**0.956**	**0.982**	**0.822**	**0.855**	**0.878**	**0.927**	**0.991**	**0.974**	**0.949**	**0.985**	**0.964**	**0.992**
glrlm_RunLengthNonUniformityNormalized	**0.783**	/	0.007	0.214	0.256	/	0.063	0.131	0.221	**0.883**	**0.761**	**0.773**	**0.981**	**0.975**	**0.989**
glrlm_ShortRunEmphasis	**0.810**	/	0.073	0.296	0.351	/	0.114	0.202	0.297	**0.887**	**0.772**	**0.770**	**0.982**	**0.974**	**0.992**
ngtdm_Coarseness	/	/	/	/	/	/	/	/	/	/	/	/	/	/	/
Total	*0.667*	*0.534*	*0.374*	*0.470*	*0.528*	*0.462*	*0.494*	*0.440*	*0.524*	*0.710*	*0.715*	*0.727*	*0.843*	*0.878*	*0.898*
Onions															
	40 vs 55	40 vs 70	40 vs 90	40 vs 120	40 vs 190	55 vs 70	55 vs 90	55 vs 120	55 vs 190	70 vs 90	70 vs 120	70 vs 190	90 vs 120	90 vs 190	120 vs 190
firstorder_Entropy	**0.896**	**0.906**	**0.914**	0.353	/	**0.963**	**0.796**	0.348	/	**0.901**	0.551	/	**0.743**	/	**0.767**
glcm_Differenceentropy	**0.998**	**0.996**	**0.995**	**0.989**	**0.989**	**0.999**	**0.997**	**0.991**	**0.991**	**0.998**	**0.996**	**0.996**	**0.997**	**0.997**	**1.000**

glcm_Jointentropy	**0.785**	**0.811**	**0.927**	0.667	0.158	**0.961**	**0.827**	0.434	/	**0.913**	0.654	/	**0.765**	/	**0.817**
gldm_DependenceEntropy	0.685	**0.830**	**0.822**	0.713	0.286	**0.940**	0.673	0.298	/	**0.858**	0.585	/	**0.792**	0.012	**0.803**
gldm_DependenceNonUniformity	**0.851**	/	0.431	0.540	/	/	**0.864**	**0.856**	/	0.666	/	**0.787**	**0.858**	0.453	/
gldm_DependenceNonUniformityNormalized	**0.897**	/	0.587	0.730	/	/	**0.868**	**0.899**	0.061	/	/	/	**0.846**	/	0.302
gldm_GrayLevelNonUniformity	0.540	0.591	0.244	0.285	0.227	0.161	/	/	/	**0.750**	0.740	0.673	**0.996**	**0.984**	**0.993**
glrlm_GrayLevelNonUniformity	0.396	0.300	/	/	**0.858**	/	/	/	/	/	/	/	**0.762**	**0.599**	**0.958**
glrlm_RunLengthNonUniformity	**0.902**	**0.953**	**0.852**	0.694	0.595	**0.938**	**0.992**	**0.925**	**0.919**	**0.930**	**0.876**	**0.784**	**0.967**	**0.958**	**0.976**
glrlm_RunLengthNonUniformityNormalized	0.736	**0.886**	**0.777**	0.003	/	**0.850**	**0.949**	0.611	**0.817**	**0.906**	0.473	0.546	**0.775**	**0.860**	**0.912**
glrlm_ShortRunEmphasis	**0.765**	**0.909**	**0.767**	/	0.150	**0.876**	**0.953**	0.647	**0.853**	**0.910**	0.485	0.647	**0.800**	**0.900**	**0.928**
ngtdm_Coarseness	/	/	/	/	/	/	/	/	/	/	/	/	/	/	/
Total	*0.704*	*0.718*	*0.665*	*0.497*	*0.408*	*0.743*	*0.792*	*0.601*	*0.607*	*0.783*	*0.596*	*0.633*	*0.775*	*0.640*	*0.769*

Bold type = ICC >0,75; / = ICC could not be calculated due to negative covariance of factors
